# The Cytomegalovirus Protein Kinase pUL97: Host Interactions, Regulatory Mechanisms and Antiviral Drug Targeting

**DOI:** 10.3390/microorganisms8040515

**Published:** 2020-04-04

**Authors:** Mirjam Steingruber, Manfred Marschall

**Affiliations:** Institute for Clinical and Molecular Virology, Friedrich-Alexander University of Erlangen-Nürnberg (FAU), 91054 Erlangen, Germany; mirjam-steingruber@gmx.net

**Keywords:** human cytomegalovirus (HCMV), protein kinase pUL97, kinase-host interactions, cyclin/cyclin-dependent kinase complexes, regulatory mechanisms, antiviral drugs

## Abstract

Human cytomegalovirus (HCMV) expresses a variety of viral regulatory proteins that undergo close interaction with host factors including viral-cellular multiprotein complexes. The HCMV protein kinase pUL97 represents a viral cyclin-dependent kinase ortholog (vCDK) that determines the efficiency of HCMV replication via phosphorylation of viral and cellular substrates. A hierarchy of functional importance of individual pUL97-mediated phosphorylation events has been discussed; however, the most pronounced pUL97-dependent phenotype could be assigned to viral nuclear egress, as illustrated by deletion of the UL97 gene or pharmacological pUL97 inhibition. Despite earlier data pointing to a cyclin-independent functionality, experimental evidence increasingly emphasized the role of pUL97-cyclin complexes. Consequently, the knowledge about pUL97 involvement in host interaction, viral nuclear egress and additional replicative steps led to the postulation of pUL97 as an antiviral target. Indeed, validation experiments in vitro and in vivo confirmed the sustainability of this approach. Consequently, current investigations of pUL97 in antiviral treatment go beyond the known pUL97-mediated ganciclovir prodrug activation and henceforward include pUL97-specific kinase inhibitors. Among a number of interesting small molecules analyzed in experimental and preclinical stages, maribavir is presently investigated in clinical studies and, in the near future, might represent a first kinase inhibitor applied in the field of antiviral therapy.

## 1. The Present Status of Controlling HCMV as a Major Human Pathogen

### 1.1. Molecular Biology of HCMV and Its Lytic Replication in Permissive Cells

HCMV, the prototypic β-herpesvirus, represents a major human pathogen and is characterized by a multifaceted mode of virus-host interaction. HCMV seroprevalence in the adult population ranges between approximately 40% to 90% and reaches even higher levels, of more than 95%, in countries with a low socio-economic standard [1]. HCMV exerts a strict species specificity and a comparably slow replication cycle spanning approximately three days in vitro [2,3]. Viral genomic DNA replication takes place in the nucleus and the double-stranded viral genome is packaged into capsids, which then undergo nuclear egress and budding through the nuclear membranes [4,5]. In the cytoplasmic virion assembly complex (cVAC), capsids are assembled with tegument proteins, before fully enveloped virus particles of approximately 150–200 nm are formed in the trans-Golgi network and released from the cell by final transition through the cytoplasmic membrane [2,6,7]. In addition to highly productive lytic infection of major target cells, such as fibroblasts, smooth muscle cells, endothelial and epithelial cells [8,9,10,11,12], HCMV causes life-long persistence by latent infection of minor target cells, such as monocytes/macrophages and CD34^+^ hematopoietic stem cells, in which latent HCMV may undergo reactivation resulting from immune insult, allogenic stimulation or differential signals (reviewed in [13]).

### 1.2. Pathogenesis of HCMV Infection

Due to the fact that primary and nonprimary infections (i.e., reactivation or reinfection) are mostly asymptomatic in healthy, immunocompetent individuals, HCMV infection usually remains clinically unrecognized. In contrast, patients with a compromised immune system, such as transplant recipients or AIDS patients, severely suffer from HCMV-related diseases, such as interstitial pneumonia, retinitis, gastroenteritis, esophagitis and organ failure, resulting in an increased mortality and morbidity [14,15,16,17]. Importantly, the immature immune system is a high risk factor for congenital cytomegalovirus infection (cCMV) of embryos and infants; thus, HCMV represents the most frequent cause for pathogen-derived developmental defects triggering mental retardation, loss of hearing or vision and microcephaly [18,19,20,21,22,23,24]. HCMV is one of few viruses that are able to cross the placenta efficiently, i.e., at least 33% of all primary infections during pregnancy of seronegative mothers, and an additional lower percentage of nonprimary infections undergo virus transmission resulting in cCMV infection of the unborn [25,26]. Thus, in Germany, approximately 3500 out of 700,000 newborns acquire cCMV per year [19]. Because of the lack of comprehensive HCMV screening, it is understood that approximately 10% of these are symptomatic at birth, including cases of stillbirth, and another 10%–15% may acquire symptoms at a later onset. HCMV can be transmitted by various body fluids, such as saliva, breast milk, vaginal secretions, semen and leukocytes containing blood and urine [27,28,29,30,31].

### 1.3. Current Options of Prevention and Control

Until today, no vaccine has been approved to control HCMV infections. Despite 60 years of intensive HCMV research, only a few antiviral drugs have been approved, which mostly interfere with the viral DNA polymerase pUL54, i.e., nucleoside/nucleotide analogs, such as the gold standard ganciclovir (GCV), its prodrug valganciclovir (VGCV), cidofovir (CDV) and the pyrophosphate analog foscarnet (FOS). Unfortunately, these drugs frequently cause severe side-effects, such as myelotoxicity, anemia and nephrotoxicity, or show poor bioavailability, which drives the selection of drug resistant virus variants [32,33,34,35,36,37]. In 2017, letermovir (LMV), the first anti-HCMV drug that targets the viral terminase complex consisting of pUL56, pUL89 and pUL51 core-subunits, was successfully assessed in clinical trials. Currently, LMV is approved for HCMV prophylaxis in hematopoietic stem cell transplantation recipients. LMV also represents a promising candidate for future combination therapies or even options of cCMV control [38,39,40,41]. However, based on the occurrence of LMV-resistant viral mutants [42] and the present lack of an approved treatment option for infants, the requirement of new antiviral drugs is still emphasized. This situation underlines the necessity of basic research to refine the understanding of the manifold and complex HCMV-host interplay and antiviral targeting strategies.

## 2. HCMV-Encoded Protein Kinase pUL97, a Multifunctional CDK Ortholog (vCDK)

### 2.1. Characteristics of the HCMV-Encoded Protein Kinase

**Figure 1:**
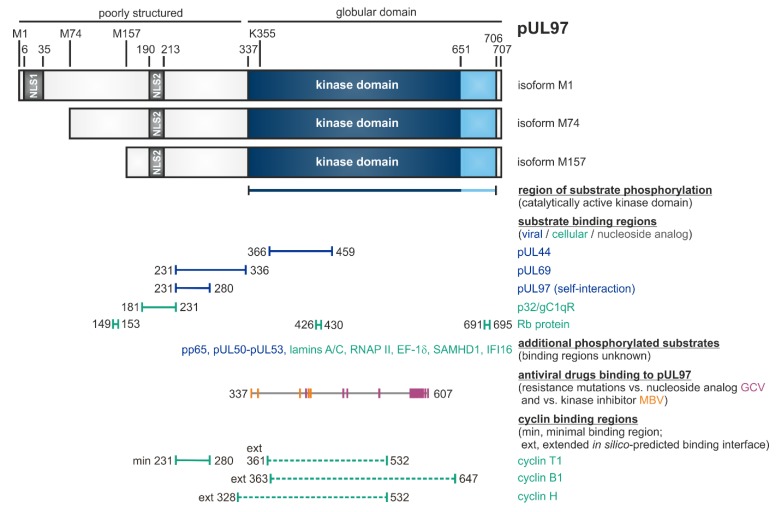
Schematic illustration of the modular structure and the so far identified binding regions within pUL97 [56]. The kinase domain is located between amino acids 337 and 706, as based on biochemical validation (or 337 and 651, as based on sequence homologies). K355 is an invariant lysine residue required for kinase activity. Expression of three pUL97 isoforms is determined by alternative translational initiation sites at M1, M74 and M157. Two nuclear localization signals (NLS1 and NLS2) are contained in the N-terminal unstructured portion of pUL97. Self-interaction/oligomerization of pUL97 is determined by amino acid region 231–280. This region overlaps with a minimal binding region for cyclin T1. Recent modeling approaches based on the in silico prediction of binding interfaces suggested extended binding interfaces for cyclins T1, B1 and H. Moreover, pUL97 is involved in the multiple regulatory steps during HCMV replication through the phosphorylation of viral and cellular substrates (see horizontal bars), as reported by several independent groups [44,45,46,54,55,57,75,79,80,81,82,83,84,94,95,96,97,98,99,100,101,122,126,127,128,129,130,131,132]. Substrates include the viral DNA polymerase cofactor pUL44, viral RNA transport factor pUL69, major tegument protein pp65, nuclear egress core protein heterodimer pUL50–pUL53, cellular multi-ligand binding protein p32/gC1qR, tumor suppressor/checkpoint protein Rb, nuclear lamins A/C, RNA polymerase II, translation factor EF-1δ, interferon-inducible proteins IFI16 and SAMHD1, as well as the therapeutically applied nucleoside analog ganciclovir (GCV; [47,56,82] and references therein). Interaction regions for GCV and the ATP-competitive pUL97 inhibitor maribavir (MBV) were defined by the location of resistance mutations detected so far (GCV: 405, 460, 466, 520, 590, 591, 592, 594, 595, 596, 597, 598, 599, 600, 601, 603, 607; MBV: 337, 353, 397, 409, 411). *Note* that this Figure represents a refined update, as adapted from an earlier version published elsewhere [56]; here, this also includes the hitherto mapped regions of resistance mutations against GCV and MBV, which possess high relevance for the discussion of an advanced antiviral drug targeting.

pUL97 is a tegument protein, which is packaged into virions and is expressed with early-late kinetics [43]. The 707-amino acid protein exists in three isoforms due to alternative initiation of translation at residues M1, M74 or M157, resulting in protein varieties of approximately 100 kDa, 80 kDa and 70 kDa, respectively (Table 1, Figure 1) [44]. The full-length kinase possesses two NLS sequences in the poorly structured N-terminus, which mediate the predominantly nuclear localization of pUL97 [45,46]. The kinase domain was assigned to the globular C-terminal part, amino acids 337–651, based on sequence homologies or extended to 337–706, based on biochemical validation [47,48,49,50]. An invariant lysine residue at position 355 is essential for kinase activity, thus leading to the catalytically inactive K355M mutant [51,52,53]. Dimers and oligomers are formed via the self-interaction domain (amino acids 231–280) of pUL97 [54]. Interestingly, the direct association of pUL97 with human cyclins has been demonstrated and, hereby, the core region responsible for cyclin T1 binding proved to be identical with the pUL97 self-interaction domain [55], thus illustrating a functional role of cyclins in pUL97 dimerization/oligomerization [52,56,57,58]. Concerning the properties of protein interaction and substrate phosphorylation of pUL97, a number of viral as well as cellular proteins have been identified thus far [see references in legend of Figure 1]. The functionality of these substrates spans various regulatory aspects of viral replication, such as nuclear egress, intrinsic immunity, genome replication and gene expression (Table 1, Figure 1). Notably, several of the pUL97-specific substrate proteins also represent substrates of cellular CDK-cyclin complexes and may thus underlie a process of dual phosphorylation through these two different kinds of protein kinases in HCMV-infected cells. While sequence conservation between the open reading frame ORF-UL97 and other kinases is generally low, functional and structural similarities have been identified between pUL97 and CDKs, so that pUL97 was termed as a multifunctional viral CDK ortholog (vCDK). Importantly, both deletion of ORF-UL97 or pharmacological inhibition of pUL97 activity resulted in a strong delay of HCMV replication [52,59,60,61], likewise explained by the fact that the kinase exerts many regulatory functions during viral replication (Table 1). On this basis, pUL97 could be validated as an interesting target for novel antiviral strategies and a panel of small molecule-type inhibitors of pUL97 activity belonging to different chemical classes has been described during the last years (see below, Section 3, Section 4, Section 5 and Section 6).

The interaction between HCMV pUL97 and human cyclins of the types B1, T1 and H has been described in our earlier reports [47,55,82]. The three cyclins obviously possess different affinities in terms of strength of pUL97 binding detected by coimmunoprecipitation (CoIP)- and mass spectrometry (MS)-based analyses. In case of cyclin B1, a requirement of catalytic activity of pUL97 for cyclin binding was identified, whereas in case of cyclin H, pUL97 interaction was found dependent on the environment of HCMV replication [82]. Recently published data indicate a substrate-bridging function of cyclin(s) for the binding of pUL97 to its substrate pp65, as determined with a pp65 mutant lacking a putative cyclin-docking motif [83].

Previous investigations led to the postulate of a substantial relevance of pUL97-cyclin interactions, as characterized by the following findings: (i) The HCMV kinase pUL97 acts as a structural CDK ortholog originally based on our bioinformatic modeling and biochemical analyses. (ii) Our initial report on pUL97-cyclin T1 interaction could be extended to additional types such as cyclins B1 and H [47,55,56,82]. (iii) The interaction pUL97-cyclins B1/T1/H was confirmed by several methods including highly sensitive mass spectrometry-based proteomics. (iv) Specifically, the interaction pUL97-cyclin B1 was found to be phosphorylation-dependent for both proteins. In addition, cyclin B1 (but not H) was phosphorylated by pUL97 in vitro [56]. (v) Using a protein assembly-based CoIP assay, the formation of binary and ternary complexes involving pUL97, cyclin H and CDK7 was identified, thus suggesting a cyclin bridging concept [125]. A central finding was that regions responsible for cyclin T1 interaction of pUL97 and pUL97-pUL97 self-interaction showed an overlap in N-terminal amino acids 231-280 (Figure 1; [54,55]). These data strongly suggest that cyclin binding is involved in pUL97-pUL97 self-interaction and very recent findings specified this activity for cyclin types T1 and H (but not B1), thus confirming the bridging function of cyclins T1/H in pUL97 dimerization or hetero-oligomerization. This self-interaction property is known to be a factor required for developing full catalytic activity of the pUL97 kinase [see references in Table 1]. The amino acid region 231–280 of pUL97 is considered as a minimal binding region for cyclin T1, which may be complemented by the additional binding of globular domain interfaces of pUL97 in the further C-terminal region, contributing to cyclin binding in a type-specific manner (cyclin T1, amino acids 361–532; cyclin B1, 363–647; cyclin H, 328–532; Figure 1; [56,82]).

In order to address the question of which spectrum of different types of human cyclins may associate with the viral pUL97 kinase, two specific experimental approaches have recently been performed. Firstly, a recombinant HCMV expressing a Flag-tagged version of pUL97 (namely the largest, fully functional isoform M1 of pUL97 encoded by HCMV AD169-UL97(Mx4)-Flag; [44]) was used for Flag-specific coimmunoprecipitation settings. The CoIP samples were then applied in a mass spectrometry-based (MS) proteomic assessment of pUL97-associated viral (Table S1) and cellular proteins (Table S2). HCMV AD169, expressing untagged pUL97, was used as a CoIP/MS specificity control. The identified viral proteins included several known interactors and/or substrates of pUL97 and showed a substantial overlap with those detected in our similar approach performed earlier, as based on the CoIP of pUL97-cyclin complexes using cyclin-specific antibodies [82]. Cellular proteins identified by this approach contained cyclins, CDKs and additional host proteins confirming earlier findings of pUL97-specific protein complexes. Notably, cyclins T1 and B1 were again safely detected, as those types of cyclins had been found by a variety of methodological approaches before (summarized in Table 2). Secondly, a panel of cyclin-specific antibodies were employed in a broader setting of CoIP analysis to learn more about the overall spectrum of pUL97-cyclin interaction. Representative members of the functional groups of cyclin types have been chosen, i.e., B-like, C-like and Y-like cyclins (Table 2, Supplementary Materials Figure S1). To this end, the cyclin-specific CoIP of pUL97 was then performed, again on the basis of total lysates prepared from HCMV-infected primary fibroblasts, followed by a quantitative assessment based on densitometric measurements (in duplicates, using two series of stained CoIP/Wb filters). The results, on the one hand, confirmed our earlier postulate that pUL97 strongly interacts with cyclin types B1, T1 and H (the latter primarily with pUL97 expressed in HCMV-infected cells, but very poorly with pUL97 transiently expressed in transfection-based settings; [56,82]). On the other hand, even more types of human cyclins could be additionally detected, either with moderate/weak (cyclins E, F and Y) or strong (cyclins B2 and K) properties of pUL97 interaction (Figure S1, Table S3 and Table 2). This topic of cyclin specificity of pUL97 and its functional relevance for HCMV replication will be further investigated by the use of recombinant HCMVs expressing mutant versions of pUL97 carrying cyclin-binding defects.

### 2.2. Phosphorylation of a Panel of Regulatory Viral Proteins and Host Factors through pUL97

**Figure 2:**
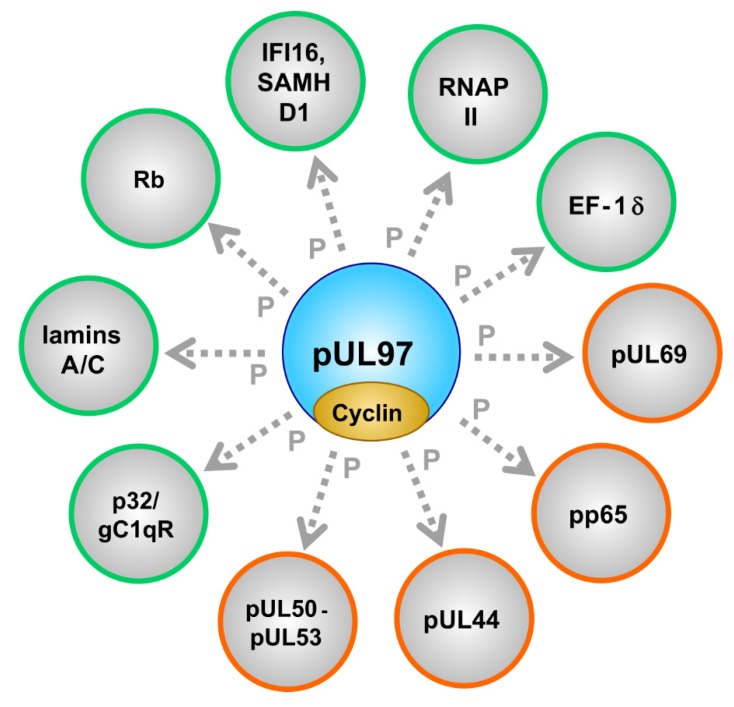
The cytomegalovirus-encoded CDK-like protein kinase pUL97 interacts with cyclins and phosphorylates a number of viral (encircled in orange) and cellular (encircled in green) substrate proteins.

Notably, pUL97 phosphorylates several viral and cellular proteins (see horizontal bars in Figure 1 for those binding regions within pUL97 that have been mapped thus far), including the viral DNA polymerase cofactor pUL44 [122], viral RNA transport factor pUL69 [79], major tegument protein pp65 [95], nuclear egress core proteins pUL50-pUL53 [99,127], cellular multiligand binding protein p32/gC1qR [98,122], tumor suppressor protein Rb [75], nuclear lamins A/C [57,84,94,98,129], RNAP II [100], translation factor EF-1δ [63,101,130], interferon-inducible, intrinsic immune restriction factors IFI16 [96] and SAMHD1 [105] (Figure 2; Table 3; compare with Tables S1–S3).

It should be emphasized that the pUL97 substrate proteins belong to several functionally different groups (Table 3), thus underlining the multifunctional nature of this singly expressed viral protein kinase. Viral proteins interacting with and being phosphorylated by pUL97 span the regulatory areas of viral nuclear egress (pUL50-pUL53 core NEC), genome replication (pUL44), tegumentation and immune-regulatory functions (pp65), viral RNA transport (pUL69) and the pUL97-pUL97 autophosphorylation/autoregulation associated with the formation of dimers and oligomers. As far as cellular substrates are concerned, the following regulatory areas are addressed: nuclear egress (lamins A/C, p32/gC1qR), cell cycle control (Rb, cyclins), intrinsic immune regulation (IFI16, SAMHD1) and transcription/translation (RNAP II, EF-1δ). The entity of this spectrum of pUL97-driven processes in virus-infected cells illustrates the functional importance of pUL97 for a high efficiency of viral replication, as demonstrated by the defects of recombinant viruses carrying UL97 deletions/mutations (up to factor 100–1000). Interestingly, the dimension of a replication defect resulting from drug-inhibited pUL97 was demonstrated to be more drastic in non-cycling compared to cycling cells [133], probably referring to the crosstalk and functional complementation between active cellular CDK-cyclin complexes and the vCDK. Moreover, the complex patterns of protein-protein interactions (PPI) undergone by pUL97 have recently been revealed by the use of highly sensitive mass spectrometry-based proteomic and phosphoproteomic approaches [56,66,82,98]. These findings make the occurrence of higher-order, pUL97-associated PPI complexes seem highly likely.

### 2.3. HCMV pUL97 and Related Herpesviral vCDKs

Most pUL97-related herpesviral kinases function as viral CDK orthologs (vCDKs). They were also termed conserved herpesviral protein kinases (CHPKs), as encoded by a gene conserved throughout the family *Herpesviridae* (e.g., prototype pUL97 and homologous kinases). Despite conservation of the UL97 gene locus, substantial variation of the primary coding sequence has been identified between herpesviruses. In addition to CHPKs, a second protein kinase is encoded by an additional non-conserved gene restricted to the subfamily α-*Herpesvirinae* (e.g., prototype pUS3 kinase of herpes simplex virus). CDK activity has been shown to be involved in multiple steps during HCMV infection [143]. vCDKs phosphorylate typical CDK substrates such as Rb and lamins A/C and show CDK activity in a yeast complementation assay [57,75,84,129]. The *Saccharomyces cerevisiae* mutant lacking activity of its sole CDK, cdc28, shows growth arrest in the early S/late G1 phase, which is overcome by CDK1 (human), pUL97 (HCMV), pU69 (HHV-6 and -7) and BGLF4 (EBV) expression [57]. In addition, pUL97 and CDK share substrate proteins, such as pUL69, RNAP II and EF-1δ [78,79,101,130]. Of note, pUL97 and CDKs phosphorylate Rb at the same residues (S780, S807, T821), leading to the inactivation of the cell cycle-inhibitory and tumor suppressor functions of Rb [75,144,145] (Table 4). In addition, the suppression of CDKs 1, 2, 5 and 9 by indirubin-derivatives increases the HCMV-inhibitory effect of maribavir (MBV), a potent pUL97 inhibitor [58]. Thus, pUL97 and CDKs possess at least partially overlapping functions.

## 3. Validation of vCDK pUL97 as an Antiviral Target and Various pUL97 Inhibitors Explored as Experimental Antiviral Drugs

### 3.1. Role of the pUL97 Kinase in Anti-HCMV Standard Therapy

The HCMV-encoded CDK ortholog pUL97 has significance in the therapy of HCMV infections, as it is responsible for the phosphorylation-mediated activation of GCV/VGCV, still representing the therapy gold standard and, similarly, additional nucleosides such as acyclovir (ACV), penciclovir (PCV) and others [69,113,174]. Hereby, the specific role of pUL97 is that nucleoside analogs have to be initially monophosphorylated in a step catalyzed by pUL97 kinase [68]. Thereafter, the active triphosphate metabolites have to be generated in a series of steps of further phosphorylation catalyzed by human guanylate kinase, dGMP kinase, phosphoglycerate kinase and potentially other host kinases [25]. In the triphosphate form, these analogs represent the active antiviral determinants, then acting as a substrate of the HCMV DNA polymerase, ultimately inhibiting the elongation of viral genome synthesis.

### 3.2. Target Validation and pUL97 Inhibitors

**Figure 3:**
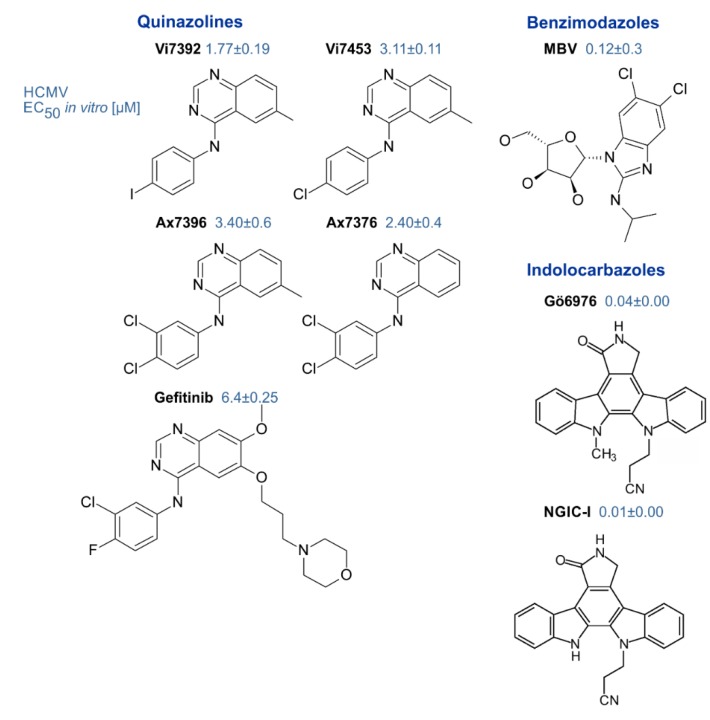
Small molecules derived from different chemical classes possessing strong anti-HCMV efficacy based on their pUL97-inhibitory potential [44,64,109,123,175,178].

Genetic mutation studies showed that pUL97 plays a rate-limiting regulatory role for the replication efficiency of HCMV and virus titers were reduced by orders of magnitude when the coding sequence was disrupted [59,61]. Moreover, pharmacological inhibition of pUL97 activity by small molecules derived from various chemical classes blocked viral replication in a manner corresponding to the pUL97 null phenotype and thus proved to be a potent antiviral targeting strategy [175]. Since then, the pharmacologic inhibition of pUL97 activity together with genetic techniques have helped to characterize the mechanisms of pUL97 supporting the viral replication and virus–host kinase interactions [52,53,56,176]. A number of inhibitors of pUL97 kinase activity have been identified that exert potent antiviral activity against HCMV [64,119,175,177]. These include indolocarbazoles [51,109,120], quinazolines [64,123,178] and benzimidazole analogs [175] (Figure 3). A number of detailed investigations, both on cell culture-based in vitro and preclinical in vivo animal models, underlined the high value of this antiviral approach (reviewed in [25,53,132,179]). Thus far, however, with the exception of maribavir, none of these compounds has progressed to clinical studies.

## 4. Clinical Investigation of the First Prototype of a Kinase Inhibitor in Antiviral Treatment: Maribavir

MBV is a benzimidazole riboside, structurally related to the terminase inhibitors BDCRB and GW275175X [25]. This molecule exerts outstanding inhibitory activity against the pUL97 kinase and shows very low levels of side/off-target effects [180]. MBV exhibits favorable pharmacokinetic properties, is well tolerated and holds promise as a new drug for the treatment of HCMV infections [181,182,183]. Thus, MBV represents a novel developmental drug that might become the first prototype of a kinase inhibitor in antiviral treatment. In the first phase III clinical study, maribavir-treated patients failed to meet the clinical endpoint objectives [184]. Currently further phase III trials are enrolling patients to compare the efficacy of MBV with GCV, and this clinical development is currently continuing (NCT02931539, NCT02927067). One limitation might be based on the fact that the inhibition of pUL97 kinase activity by MBV interferes with the activation of GCV, thus resulting in drug antagonism, which most probably reduces their antiviral efficacies in a combination therapy [124,185,186]. Mutations conferring MBV resistance are distinct from those conferring GCV resistance, with sites of mutations partly located outside the conserved kinase domains [114,132,187]. In rare cases, kinase domain mutations arise in the laboratory that are essentially kinase null mutations and can confer resistance to MBV or GCV [179]. Notably, however, MBV exerts activity against typical GCV-resistant strains and might therefore create new options in the treatment of drug-resistant HCMV infections [175,188,189]. Interestingly, the three different isoforms of the kinase also show altered susceptibility of the virus to MBV [44]. An additional type of an intermediate-level MBV-resistance has been identified for viral variants carrying mutations, not in the UL97 but rather in the UL27 gene [190,191]. To date, it is not clear whether resistance mutations in UL27 would arise in clinical settings, since in animals the deletion of ORF-UL27 resulted in a modest half-log reduction in viral in vitro replication capacity, with no apparent effect on replication in vivo [192].

## 5. The Relevance of Targeting a Herpesviral Kinase Activity in Antiviral Strategies

The HCMV-encoded kinase pUL97 combines two different aspects of medical importance, namely serving as promoter of prodrug activation through the activating monophosphorylation of GCV, VGCV and related nucleoside analogs and as a validated target of antiviral kinase inhibitors. The currently ongoing clinical investigations of MBV are approaching an exciting interim phase and it will be highly relevant to see whether this drug candidate achieves primary endpoints. MBV might not only represent a novel drug for the treatment and prevention of HCMV disease but it would likewise be a very promising novel prototype of a kinase inhibitor that might—compared to the numerous currently approved kinase inhibitors in antitumoral treatments—for the first time enter the field of antiviral therapy. Notably, the applicability of a further mode of action of antiviral drugs would directly broaden the options of overcoming previous problems with antiviral drug resistance. The pharmacological interference with viral kinase activity/protein phosphorylation by MBV, in addition to the targeting of viral genome replication/polymerase activity (GCV) and viral terminase activity/genome processing (LMV), would open a third mechanistic option of HCMV treatment. Thus, resistant mutants arising from GCV and LMV treatment would very probably remain susceptible to MBV treatment, so that variable regimens might become available, possibly including combination therapies. It should be mentioned, however, that GCV and MBV combination would underlie an antagonistic principle, due to the two counteractive roles of pUL97 in such a case (prodrug converting GCV phosphorylation through active pUL97 versus an inhibition of pUL97 activity by MBV). Nevertheless, other combinations between MBV and LMV, GCV and LMV or the involvement of additional approved anti-herpesviral drugs, such as CDV, ACV etc., might lead to a substantial improvement of medication regimens. In this sense, anti-HCMV therapy might also greatly benefit from the experiences made in the field of human immunodeficiency virus/AIDS during the past decades, as mostly gathered by the steady development of novel antiretroviral combination therapies.

## 6. Future Perspectives of Novel Mechanistic Options of pUL97-Specific Drug Targeting

It should also be stressed that the drug targeting of a viral kinase such as pUL97 may not exclusively be limited to classical ATP-competitive types of kinase inhibitors including MBV. This strategy entails also untypical, thus far therapeutically untapped possibilities of kinase targeting, i.e., non-ATP-competitive modes of targeting [193,194,195]. It is quite conceivable that additional research work may reveal prototypes of non-ATP-competitive substrate inhibitors of pUL97 that could be directed to blocking the phosphorylation of individual pUL97 substrates, without inactivating the functionality of the pUL97 kinase domain. Such types of kinase inhibitory small molecules can either function through a shielding mechanism directed at one or several defined phosphorylation sites of a pUL97 substrate (phosphosite inhibitors) or it might cause a steric hindrance of pUL97 substrate recognition (allosteric assembly blockers of kinase-specific protein complexes, including an interference with pUL97-cyclin association [56,196,197]). Even the involvement of covalent binders appears within the realms of possibility. Recently, remarkable progress has been reported in the field of generating small molecules acting as covalent kinase binders with selectivity to the tumor-relevant mutant G12C of the human KRAS tyrosine kinase [198]. The kinase inhibitor AMG510 has recently been successfully investigated in clinical stage I/II [199]. Combined, the increase in understanding of the individual molecular features and the overall functionality of pUL97, together with the development of a number of highly interesting and innovative small molecule-type kinase inhibitors, nourishes the long-held optimism about translational success with pUL97 inhibitors in the near future. Thus, one of the experimentally and pharmacologically approved inhibitors, such as maribavir or, alternatively, cancer-approved CDK inhibitors, represent the first candidates of kinase inhibitor to be clinically applied in antiviral therapy.

## Figures and Tables

**Table 1:** Characterization of the molecular features and functional properties of the HCMV protein kinase pUL97.

Property	General Description	Specific Feature	Own Findings (MM lab.)	Various References
**Type of kinase**	Ser/Thr	target site P + 5, target site LxSP	[51,53,56,62,63,64]	[65,66,67,68,69]
**Molecular mass, basic features**	100/80/70 kDa	isoforms due to alternative translational start sites	[44,45,54]	[43,52,67]
**Expression pattern**	three isoforms M1, M74, M15 (referring to other herpesviral protein isoforms)	differences in substrate binding, nuclear translocation and drug susceptibility	[44,70]	[71,72,73,74]
**Similarity and sequence conservation with other kinases**	low	<35% identity with herpesviral kinases, <15% identity with cellular kinases	[45,63]	[48,49,50,75]
**Sequence conservation ORF-UL97 of HCMVs**	high	no variation of translational start sites, NLS sequences or kinase domains	[44]	[76,77]
**Related to cell kinases**	cyclin-dependent kinases (CDKs), viral CDK ortholog	functional overlap with CDKs, specific crosstalk with CDK9, CDK7 and CDK1, direct interaction with cyclins	[47,55,56,78,79,80,81,82,83]	[57,84,85]
**Coregulation of viral replication by pUL97 and cellular kinases**	several novel cellular kinases, including CDKs, identified to be involved in HCMV replication	virus-supporting functions in signaling pathways and nuclear capsid egress	[55,56,86,87]	[88,89,90,91,92,93]
**Substrate proteins**	viral, cellular	pUL44, pUL69, pp65, Rb, p32/gC1qR, nuclear lamins, EF-1δ, RNAP II, IFI16, SAMHD1	[53,79,87,94,95,96,97,98] (references therein)	[57,75,84,99,100,101,102,103,104,105,106] (see also refs. in Figure 1)
**Involvement in intrinsic immunity evasion**	stimulation of viral counterdefense of immunity	interaction with cellular restriction factors IFI16 and SAMHD1	[96,107]	[108]
**Auto-phosphorylation**	pronounced auto-phosphorylation activity, several N-terminal Ser and Thr residues	autophosphorylation most probably required for kinase activity/autoactivation	[44,54,56,94,109]	[65,66,110]
**Nucleoside phosphorylation**	ganciclovir, valganciclovir, penciclovir, acyclovir, etc.	prodrug-activating monophosphorylation as an essential step in antiviral therapy	[51,111]	[59,112,113,114,115]
**Incorporation into virions**	component of virion tegument	virion-derived pUL97 possesses highly detectable kinase activity	[45,95]	[43,116,117]
**Intracellular localization**	mainly nuclear	two nuclear localization signals, NLS-1 (6–35), NLS-2 (164–213), classical importin-α pathway	[45,46,63,97,118]	[60]
**Inhibitors of pUL97**	small molecules (<500 Da, various chemical classes)	indolocarbazoles, benzimidazoles, quinazolines, others	[53] (references therein) [44,64,119]	[114,120,121]
**Phenotype of pUL97 inhibition or UL97 deletion**	strongly reduced viral replication efficiency (100–1000-fold)	delayed replication kinetics; impaired genomic replication; impaired viral nuclear egress	[44,51,53,94,109,122,123]	[59,61,104,124]

**Table 2:** Summarized findings of pUL97-cyclin interaction derived from complementary experimental settings *.

	A HCMV-Infected Cells						B Recombinant Expression		
	Cyclin Types	Cyclin IP MS|Wb		pUL97 IP MS|Wb		Colocalization in IF	Transfection	Yeast Two-Hybrid System	Phosphorylation by pUL97 in IVKA
**B-like**	Cyclin A	+	±	-	-	..	..	..	..
	**Cyclin B1**	+	+	+	-	+	+	..	+
	Cyclin B2	-	+	-	-	-	..	..	..
	Cyclin D1	-	-	-	-	-	-	..	..
	Cyclin E	±	±	-	-	..	..	..	..
	Cyclin F	..	±	-	-	..	..	..	..
**C-like**	**Cyclin H**	+	+	-	-	+	-	-	-
	Cyclin K	..	+	-	-	..	..	..	..
	Cyclin L2a	*..*	-	-	-	..	..	..	..
	**Cyclin T1**	+	+	+	+	+	+	+	-
**Y-like**	Cyclin Y	..	±	-	-	..	..	..	..

* Data on pUL97-cyclin interaction were derived from the experimental settings of either mass spectrometry-based proteomics (MS) or Western blot detection (Wb), both performed by the use of coimmunoprecipitates derived from cyclin-specific immunoprecipitation (cyclin IP) or pUL97 immunoprecipitation (pUL97 IP). Colocalization patterns between pUL97 and individual cyclins, in particular nuclear punctate patterns of accumulation in viral replication centers, were determined by indirect immunofluorescence (IF) double-staining and confocal imaging. Recombinant expression of pUL97 and/or cyclins was performed by transient transfected of 293T cells (transfection), yeast cells (yeast two-hybrid assay) or bacterial expression systems, the latter for analyzing the phosphorylation of recombinant cyclins by transfection-derived pUL97 in the in vitro kinase assay (IVKA). In panel A, the criteria of categorization were set as follows: +, strong pUL97-cyclin interaction (MS: WSC ≥ 4; Wb: % IP values > 20% IP control and ≥ 15-fold above Flag neg. control); ±, weak interaction (MS: WSC = 3; Wb: % IP values > 20% IP control or ≥15-fold above Flag neg. control); -, no detectable interaction; .., not determined. Note that the combined experimental data provide strongest evidence for cyclins B1, H and T1 to represent the major cyclin types interacting with pUL97, as highlighted by bolt print.

**Table 3:** Characteristics of viral and cellular substrate proteins of the HCMV vCDK pUL97 as well as pUL97-associated cyclins.

Protein Origin	Designation	Function	Remarks	References
**Viral**	pUL50	core nuclear egress protein (NEC)	forms the NEC groove, multiple PPIs, phosphorylated by viral and cellular kinases	[62,99,126,127,134,135]
**Viral**	pUL53	core nuclear egress protein (NEC)	forms NEC hook, possibly docking to capsids, phosphorylated by viral kinase	[99,136,137]
**Viral**	pUL44	DNA polymerase pUL54 processivity factor	phosphorylation might regulate activity	[104,122]
**Viral**	pp65	major tegument protein	massively phosphorylated and virion-associated with pUL97	[44,60,95]
**Viral**	pUL69	RNA transport regulator	phosphorylation regulates activity	[78,79,138,139]
**Viral**	pUL97	CDK-like serine/threonine protein kinase, multifunctional	dimers/oligomers, autophosphorylation	[50,53,54,65,110,114,132]
**Cellular**	p32/gC1qR	multiligand binding protein, multifunctional	NEC bridging factor	[94,98,140]
**Cellular**	lamins A/C	structural and regulatory components of the nuclear envelope	lamin phosphorylation is a rate-limiting step of viral nuclear egress	[57,84,94,97,129,141]
**Cellular**	Rb	retinoblastoma protein, cell cycle check-point regulator	multiply phosphorylated by CDKs and pUL97	[48,57,85,103,106]
**Cellular**	IFI16 and SAMHD1	intrinsic immune restriction factors of virus infections	interferon-induced, phosphorylation-controlled	[96,105,107,108,142]
**Cellular**	RNAP II	main cellular mRNA transcriptase	activity-regulated by C-terminal phosphorylation (CTD)	[59,61,90,100]
**Cellular**	EF-1	translation elongation factor 1 delta	activity-regulated by phosphorylation	[53,101]
**Cellular**	cyclins	regulatory subunits of CDKs	types B1, H, T1 were found pUL97-associated (possibly also B2, K, others)	[55,56,58,84,92]

**Table 4:** Comparison of distinct molecular characteristics shared between vCDK pUL97 and human CDKs.

Kinase Characteristics	pUL97	CDK1	CDK7	CDK9
**Amino acids (aa)**	707	297	345	372
**Aa sequence identity to pUL97**	100%	4.5%	4.2%	8.6%
**Cyclin binding partner [56,146,147]**	cyclin B1 cyclin H cyclin T1	cyclin A1/A2 cyclin B1/B2/B3 cyclin D1/D3 cyclin F cyclin K (activating)	cyclin H cyclin A2 cyclin B1/B2 cyclin E (activating)	cyclin T1/T2 cyclin H cyclin K (activating)
**Region in the kinase required for cyclin binding [55,148,149]**	cyclin T1: ^231^ESQDSAVASGPGRIPQPLSGSSGEESATAVEADSTSHDDVHCTCSNDQII^280^ and *in silico*-predicted binding interfaces for cyclins B1, H and T1 spanning aa 328–647	cyclin B1: a positively charged region in the N-lobe (containing K6, K9, K34, R36, R75, excluding the PSTAIRE helix) cyclin A2: ^45^PSTAIRE^51^	cyclin H: ^56^NRTALRE^62^	cyclin T1/T2, K: ^60^PITALRE^66^
**Cyclin phosphorylation [56,82,146,150,151,152,153]**	cyclin B1	cyclin B1 S126 by CDK1 S128 by CDK1	cyclin H by CDK7/CDK8-cyclin C (inhibitory)	n.d.*
**T-loop phosphorylation [56,154,155,156,157,158,159,160,161]**	no, (possibly S483)	T161 by CAK (activating)	S164 and T170 by CDK1/CDK2 (activating)	T186 by CaMK1D or CDK9 (S175 by CAK, not essential for activity)
**Autophosphorylation [110,155,156]**	yes	no	(yes) outside the T-loop	yes within the T-loop
**Rb phosphorylation [66,75,82,145,162,163**]	S780, S807, S811, T821, T823, T826	S249, T252, T373, S807, S811	no	C-terminus (793–834)
**p53 phosphorylation [164,165,166]**	*n.d.*	S315	S33 (MAT1-dependent)	S33, S315, S392
**Lamin A/C phosphorylation [84,141,167,168]**	S22 (inhibitory)	S22, S392 (inhibitory)	no	no
**CTD RNAP II phosphorylation [100,169,170]**	S2, S5 (activating)	no	S2, S5, S7 (activating)	S2, S5, S7 (activating)
**SAMHD1 phosphorylation [171,172,173]**	yes	T592	*n.d.*	*n.d.*
**HCMV pUL69 phosphorylation [78,79]**	yes	yes	yes	yes
**HCMV pUL50 phosphorylation [127]**	yes	yes	*n.d.*	*n.d.*

* n.d., not determined.

## Supplementary Materials

The following are available online at https://www.mdpi.com/2076-2607/8/4/515/s1.
