# Supplementary material for: The Cytomegalovirus Protein Kinase pUL97: Host Interactions, Regulatory Mechanisms and Antiviral Drug Targeting
Source: Microorganisms. 2020 Apr 4;8(4):515. doi: 10.3390/microorganisms8040515 (PMC7232230; doi:10.3390/microorganisms8040515)
Supplement: Supplementary file 1 [file microorganisms-08-00515-s001.pdf]

microorganisms

Supplementary materials

Review

The cytomegalovirus protein kinase pUL97: host interactions, regulatory mechanisms and antiviral drug targeting

Mirjam Steingruber[1] and Manfred Marschall[1,]*

[1]Institute for Clinical and Molecular Virology, Friedrich-Alexander University of Erlangen-Nürnberg (FAU), Erlangen, Germany

***Correspondence: manfred.marschall@fau.de; Tel.: +49-9131-85-26089

microorganisms

MDPI

Table S1. Viral proteins coimmunoprecipitated in complex with pUL97-Flag using lysates of HCMV-infected HFFs *

		IP: mAb/pAb-Flag					
		pUL97-Flag					
**Gene**	**Description**	**I**	**II**	**III**	**IV**	**mean**	**pUL97**
**UL83[1]**	**65 kDa phosphoprotein pp65**	**584**	**611**	**403**	**458**	514	13
**UL97[1]**	**Serine/threonine protein kinase UL97**	**141**	**105**	**170**	**173**	147	-
UL25	Phosphoprotein 85	**98**	**81**	**67**	**73**	80	3
MCP	Major capsid protein	**78**	**37**	**59**	**65**	60	4
RIR1	Ribonucleoside-diphosphate reductase large subunit-like protein	**58**	**56**	**45**	**49**	52	b.c.
**UL44[1]**	**DNA polymerase processivity subunit**	**59**	**41**	**44**	**51**	49	5
UL32	Large structural phosphoprotein	**54**	**23**	**37**	**47**	40	-
IRS1	Protein IRS1	**36**	**26**	**29**	**24**	29	b.c.
UL48	Large tegument protein deneddylase	**23**	**15**	**35**	**44**	29	-
UL47	Inner tegument protein	**27**	**13**	**21**	**29**	23	-
TRS1	Protein HHLF1	**24**	**18**	**21**	**24**	22	b.c.
UL35	Protein UL35	**23**	**24**	**17**	**14**	20	-
TRX2	Triplex capsid protein 2	**21**	**9**	**15**	**18**	16	-
**NEC2[1]**	**Nuclear egress protein 2 pUL50**	**18**	**13**	**17**	**13**	15	b.c.
UL26	Tegument protein UL26	**13**	**11**	**17**	**13**	14	-
UL112/UL113	Early phosphoprotein p84	**7**	**9**	**18**	**17**	13	-
UL82	Protein pp71	**15**	**4**	**14**	**13**	12	-
UL104	Portal protein	**14**	**13**	**9**	**8**	11	-
DBP	Major DNA-binding protein	**24**	**5**	**5**	**8**	11	-
UL84	Protein UL84	**8**	**5**	**10**	**14**	9	-
UL43	Tegument protein UL43	**14**	**5**	**3**	**8**	8	-
TRX1	Triplex capsid protein 1	**12**	**3**	**6**	**7**	7	-
CVC1	Capsid vertex component 1	**8**	**7**	**6**	**6**	7	-
**NEC1[1]**	**Nuclear egress protein 1 pUL53**	**7**	**5**	**8**	**5**	6	b.c.
UL29	Protein UL29/UL28	**5**	**5**	b.c.	**4**	5	-
US22	Early nuclear protein HWLF1	**8**	**9**	b.c.	**5**	7	b.c.
**UL69[1]**	**mRNA export factor ICP27 homolog**	**52**	**37**	**24**	**27**	35	8
IR11	Viral Fc-gamma receptor-like protein IR11	**15**	**9**	**9**	**6**	10	b.c.
UL98	Alkaline nuclease	**4**	**3**	**7**	**6**	5	-
UL54	DNA polymerase catalytic subunit	**3**	-	**10**	**4**	6	-
UL103	Cytoplasmic envelopment protein 1	**4**	b.c.	b.c.	b.c.	4	-
UL24	Protein UL24	**4**	b.c.	**4**	**4**	4	-
UL70	DNA primase	**3**	b.c.	b.c.	-	3	-
UL122	Viral transcription factor IE2	**4**	b.c.	**6**	**5**	5	-
TRM3	Tripartite terminase subunit 3	b.c.	b.c.	**4**	**3**	4	-
UL94	Cytoplasmic envelopment protein 2	-	b.c.	**4**	b.c.	4	-
US20	Membrane protein US20	b.c.	b.c.	**3**	b.c.	3	-
CVC2	Capsid vertex component 2	b.c.	-	b.c.	**3**	3	-

*HFFs were infected with HCMV AD169-UL97(Mx4)-Flag (expressing the large isoform M1 of pUL97 C-terminally tagged with Flag) and harvested 4 d p.i. for immunoprecipitation (IP) of protein complexes and their subsequent analysis by mass spectrometry. The four columns I-IV present the number of peptides identified in four replicates. Flag-specific monoclonal and polyclonal antibodies were used for IP as indicated. All WSC values were corrected against the negative control value obtained by IP with a rabbit Fc fragment. Flag-specific IP using lysates from HCMV AD169-infected cells expressing untagged pUL97 served as an additional negative control. For a description of the methods for the proteomic analysis, see previous reports on similar investigations [51, 77]. Cut-off criteria were chosen as follows: (i) ≥ 20% IP control and (ii) > 15-fold above Flag neg. control, in either of the two samples pUL97-Flag or pUL97. [1]Known interactors and/or phosphorylated substrates of pUL97 in bold letters; b.c., below cut-off (cut-off ≥ 3 WSC); WSC, weighted spectral counts.

microorganisms

MDPI

Table S2. Cyclins, CDKs and additional cellular proteins in complex with pUL97-Flag using lysates of HCMV-infected HFFs: the selection of scored proteins was performed according to their previously published properties of putative pUL97 interaction*

		IP: mAb/pAb-Flag					
		pUL97-Flag					
**Gene**	**Description**	**I**	**II**	**III**	**IV**	**mean**	**pUL97**
LMNA[3]	Prelamin-A/C	71	58	22	26	44	6
**IFI16[1]**	**Interferon gamma-inducible protein 16**	10	10	9	7	9	-
CDC5L[3]	Cell division cycle 5-like protein	4	4	4	5	4	-
EMD[2]	Emerin	28	18	4	b.c.	17	b.c.
**LMNA[1]**	**Lamin A/C**	9	5	3	b.c.	6	b.c.
LRRFIP2[3]	Leucine-rich repeat flightless-interacting protein 2	6	6	b.c.	-	6	-
RBL1[3]	Retinoblastoma-like protein 1	6	1	10	7	6	-
LEMD3[3]	Inner nuclear membrane protein Man1	3	-	-	-	3	-
**CDK1[1]**	**Cyclin-dependent kinase 1**	b.c.	b.c.	15	8	11	-
**CCNT1[1]**	**Cyclin T1**	-	b.c.	4	3	4	-
**RB1[1]**	**Retinoblastoma-associated protein**	b.c.	b.c.	3	b.c.	3	-
**CCNB1[1]**	**Cyclin B1, G2/mitotic-specific cyclin**	-	-	4	3	4	-
**CDK9[1]**	**Cyclin-dependent kinase 9**	b.c.	b.c.	3	b.c.	3	-
CDC20[3]	Cell division cycle protein 20 homolog		b.c.	4	3	4	-
PPIB[3]	Peptidyl-prolyl cis-trans isomerase B	b.c.	b.c.	b.c.	32	32	b.c.
FKBP3[3]	Peptidyl-prolyl cis-trans isomerase FKBP3	-	-	-	8	8	-
CDK5RAP2[3]	CDK5 regulatory subunit-associated protein 2	-	-	b.c.	3	3	-

*HFFs were infected with HCMV AD169-UL97(Mx4)-Flag (expressing the large isoform M1 of pUL97 C-terminally tagged with Flag) and harvested 4 d p.i. for IP of protein complexes and their subsequent analysis by mass spectrometry. The four columns I-IV present the number of peptides identified in four replicates. Flag-specific monoclonal and polyclonal antibodies were used for IP as indicated. All WSC values were corrected against the negative control value obtained by IP with a rabbit Fc fragment. Flag-specific IP using lysates from HCMV AD169-infected cells expressing untagged pUL97 served as an additional negative control. For a description of the methods for the proteomic analysis, see previous reports on similar investigations [51, 77]. Cut-off criteria were chosen as follows: (i) ≥ 20% IP control and (ii) > 15-fold above Flag neg. control, in either of the two samples pUL97-Flag or pUL97. [1]Known interactors and/or phosphorylated substrates of pUL97 in bold letters; [2]known components of pUL97-associated complexes; [3]putatively associated proteins; b.c., below cut-off (cut-off ≥ 3 WSC); WSC, weighted spectral counts.

microorganisms

MDPI

Table S3. Quantities of pUL97 detected by cyclin-specific coimmunoprecipition analysis (CoIP/Wb)*

Cyclin type	pUL97-Flag	pUL97 % IP	Flag neg.	pUL97-Flag	pUL97 x-fold Flag neg.	Flag neg.
Cyclin B1	20.7	1.3	0.8	27.3	1.7	1.0
Cyclin B2	16.3	40.5	0.6	28.6	71.0	1.0
Cyclin D1	8.9	11.2	4.9	1.8	2.3	1.0
Cyclin F	7.1	24.3	14.0	0.5	1.7	1.0
Cyclin K	20.9	23.7	-1.5	20.9	23.7	1.0
Cyclin L2a	5.4	16.7	1.4	3.8	11.7	1.0
Cyclin Y	6.0	18.5	-0.5	6.0	18.5	1.0

*Signal intensities of CoIP/WB experimentation (partly depicted by Figure S1) were quantitated densitometrically using Aida Image Analyzer v.4.23 (mean values of densitometrical dermination in quadruplicate, i.e. performing quantitation duplicates each of two independent Wbs). Direct pUL97-Flag IP via Flag-antibody was set to 100% and values corrected against the rabbit Fc fragment negative control by subtraction. Flag-specific IP using lysates from HCMV AD169-infected cells expressing untagged pUL97 (Flag neg.) served as an additional negative control. Cut-off criteria were chosen as follows: (i) IP % values >20% of IP control (positive, +) or value 15-20% of IP control (slightly positive, ±) and >15-fold above Flag neg. control, at least in one of the samples pUL97-Flag or pUL97.

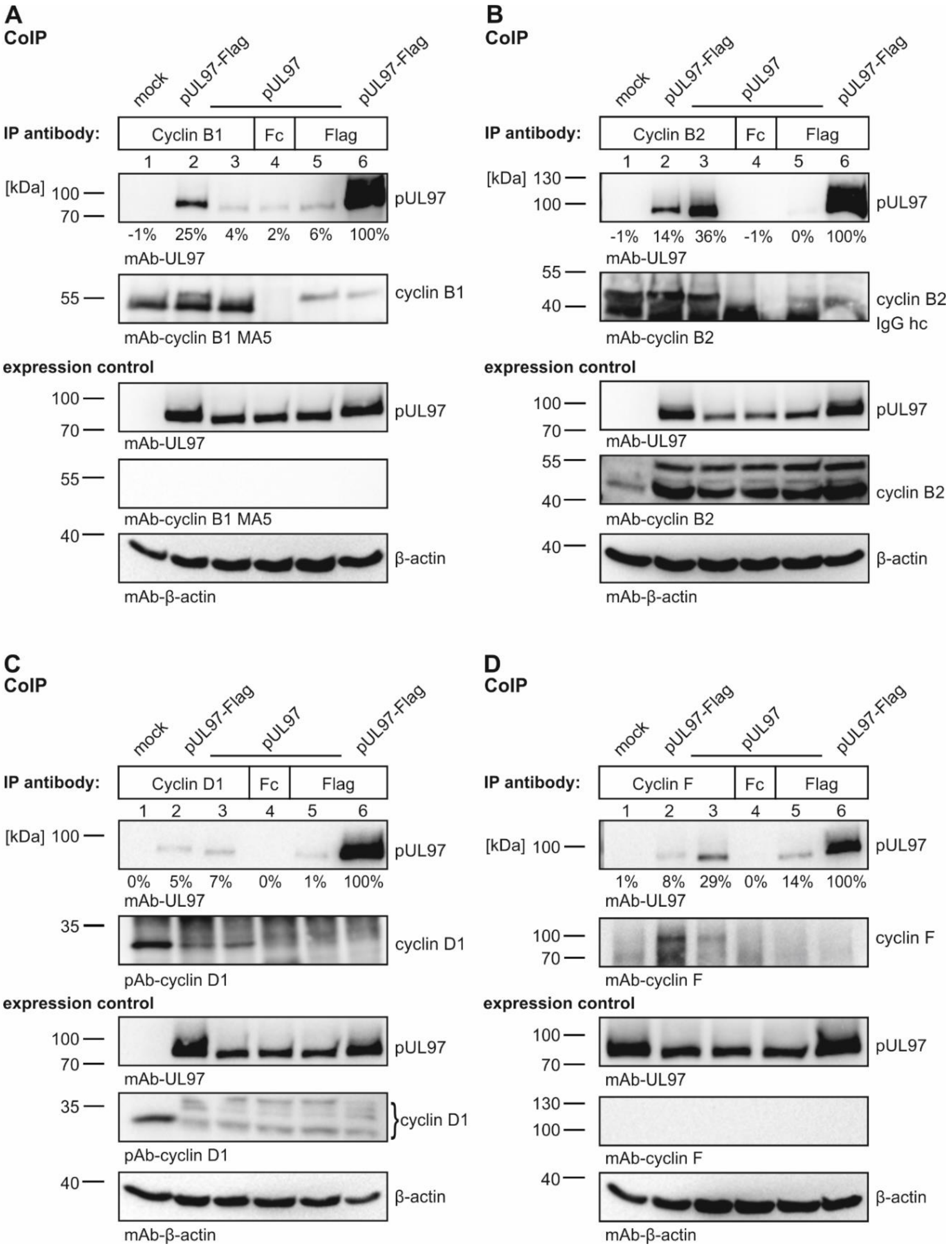
A
CoIP
mock
pUL97-Flag
pUL97
pUL97-Flag
IP antibody:
Cyclin B1
Fc
Flag
1 2 3 4 5 6
[kDa] 100
70
pUL97
-1% 25% 4% 2% 6% 100%
mAb-UL97
55
cyclin B1
mAb-cyclin B1 MA5
expression control
100
70
pUL97
mAb-UL97
55
mAb-cyclin B1 MA5
40
β-actin
mAb-β-actin
B
CoIP
mock
pUL97-Flag
pUL97
pUL97-Flag
IP antibody:
Cyclin B2
Fc
Flag
1 2 3 4 5 6
[kDa] 130
100
pUL97
-1% 14% 36% -1% 0% 100%
mAb-UL97
55
40
cyclin B2
IgG hc
mAb-cyclin B2
expression control
100
70
pUL97
mAb-UL97
55
40
cyclin B2
mAb-cyclin B2
40
β-actin
mAb-β-actin
C
CoIP
mock
pUL97-Flag
pUL97
pUL97-Flag
IP antibody:
Cyclin D1
Fc
Flag
1 2 3 4 5 6
[kDa] 100
pUL97
0% 5% 7% 0% 1% 100%
mAb-UL97
35
cyclin D1
pAb-cyclin D1
expression control
100
70
pUL97
mAb-UL97
35
cyclin D1
pAb-cyclin D1
40
β-actin
mAb-β-actin
D
CoIP
mock
pUL97-Flag
pUL97
pUL97-Flag
IP antibody:
Cyclin F
Fc
Flag
1 2 3 4 5 6
[kDa] 100
pUL97
1% 8% 29% 0% 14% 100%
mAb-UL97
100
70
cyclin F
mAb-cyclin F
expression control
100
70
pUL97
mAb-UL97
130
100
mAb-cyclin F
40
β-actin
mAb-β-actin

microorganisms

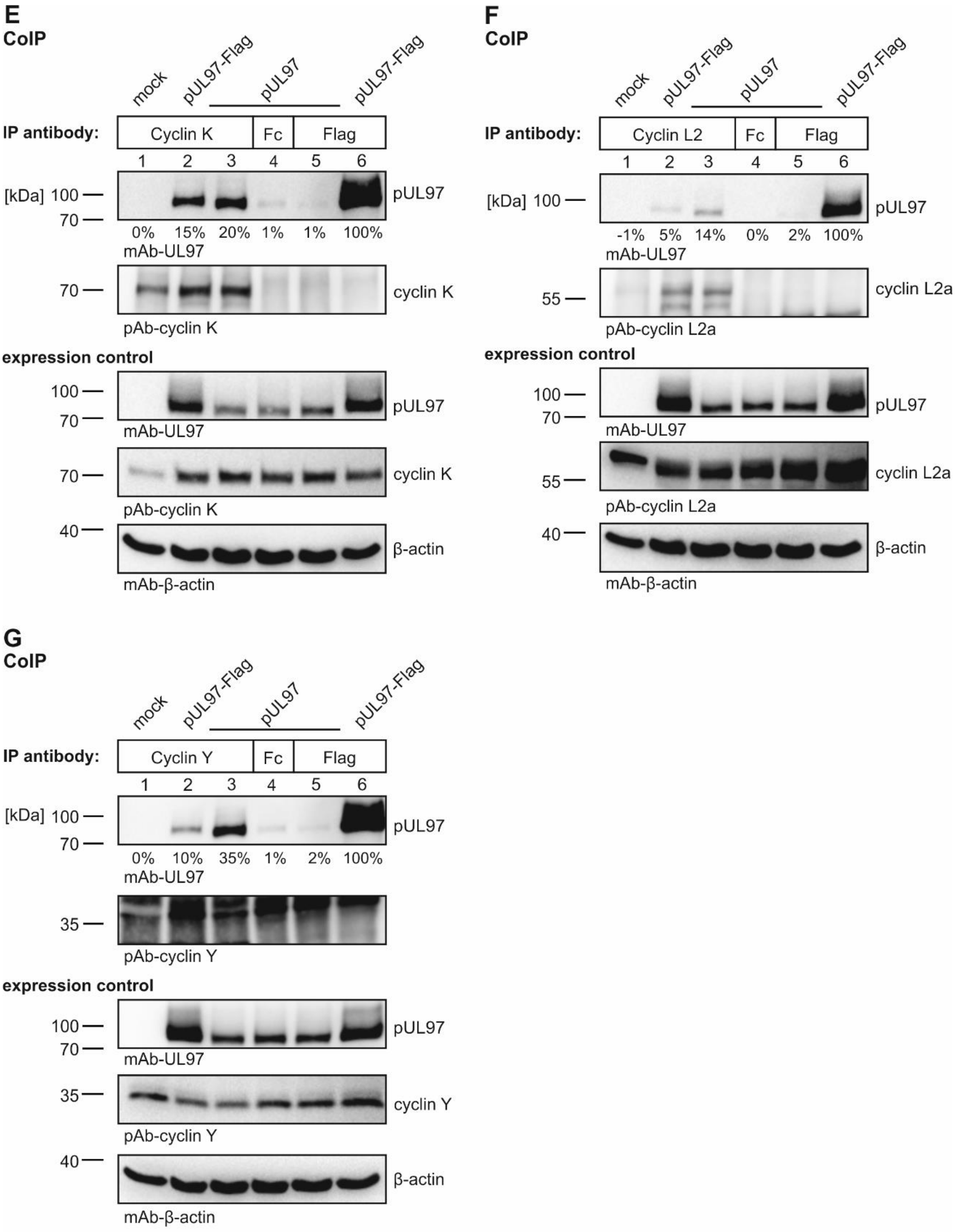

Figure S1. Representative series of primary data of the CoIP/Wb analysis of pUL97-cyclin interactions (see Table S3 for densitometrical quantitation and Table 2 presenting summarized data of various determinations of pUL97-cyclin interaction). HFFs were infected with HCMV strain AD169 (expressing untagged pUL97) or the recombinant virus AD169-UL97(Mx4)-Flag (expressing the large isoform M1 of pUL97 C-terminally tagged with Flag). CoIPs were performed with a specificity of the individually used IP antibodies as indicated, **(A)** cyclin B1, **(B)** cyclin B2, **(C)** cyclin D1, **(D)** cyclin F, **(E)** cyclin K, **(F)** cyclin L2a and **(G)** cyclin Y. Fc, antibody Fc fragment used as a negative control.
